# Pathogenic role of mitochondrial DNA mutations in heart failure: clinical features, mechanisms, and therapeutic prospects

**DOI:** 10.3389/fcvm.2026.1781927

**Published:** 2026-04-01

**Authors:** Mingyang Ni, Aijia Zheng, Hang Zheng, Wenqing Jia, Yuansheng Wang, Yuqing Peng, Chenguang Yao, Yanhong Wei, Xueli Wang, Sini Kang

**Affiliations:** 1 Cardiodynamics and Assistive Tech Group, National “111” Center for Cellular Regulation and Molecular Pharmaceutics, Wuhan Joint Laboratory (China-Serbia) of Biomedical and Health Science, School of Life and Health Sciences, Hubei University of Technology, Wuhan, China; 2 Hubei Provincial Enterprise Technology Center, Wuhan Vickor Medical Technology Co., Ltd., Wuhan, China

**Keywords:** cardiomyopathy, energy metabolism, heart failure, immune activation, mitochondrial DNA mutations, mitochondrial dysfunction, oxidative stress, therapeutic strategies

## Abstract

Heart failure (Heart failure, HF) is a complex clinical syndrome caused by any abnormality in the structure or function of the heart, resulting in impaired ventricular filling or ejection capacity, with mitochondrial dysfunction recognized as one of the key pathological foundations. In recent years, numerous studies have demonstrated that mitochondrial DNA (mtDNA) mutations play a significant role in cardiomyopathy and HF; however, systematic understanding of their modes of action in disease progression remains limited. Most studies have attributed the pathogenic effects of mtDNA mutations to impaired energy metabolism, emphasizing the consequences of defective oxidative phosphorylation and insufficient ATP production on myocardial function. Emerging evidence, however, indicates that mtDNA mutations also contribute to the development and progression of HF by inducing reactive oxygen species accumulation, disrupting mitochondrial structural and dynamic homeostasis, and activating innate immune inflammatory signaling pathways. Furthermore, variations in mtDNA mutation load and heteroplasmy levels constitute an important molecular basis for the diverse clinical phenotypes of HF, although the underlying mechanisms have yet to be systematically integrated. This review comprehensively summarizes the pathogenic mechanisms of cardiac mtDNA mutations and their heteroplasmy in HF, with particular emphasis on the intrinsic links among mitochondrial metabolic reprogramming, oxidative stress, immune activation, and myocardial remodeling, and outlines potential diagnostic and therapeutic strategies based on mitochondrial dysfunction and mtDNA stability.

## GRAPHICAL ABSTRACT

**Figure:**
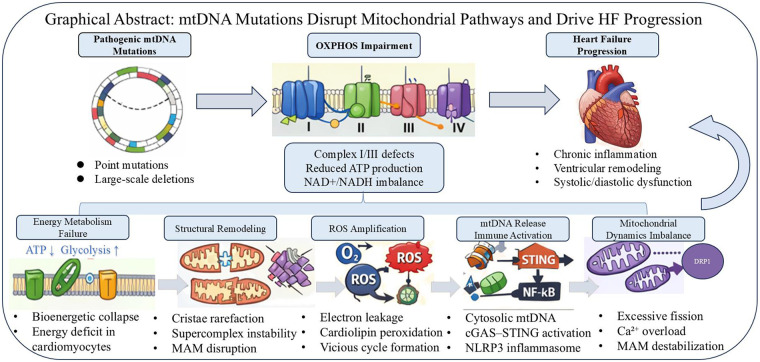
mtDNA Mutations Disrupt Mitochondrial Pathways and Drive Heart Failure Progression. This illustrated summary outlines a causal framework for the progression of cardiac dysfunction and heart failure caused by pathogenic mitochondrial DNA (mtDNA) mutations. Pathogenic mitochondrial DNA mutations (e.g., point mutations or large deletions) impair the assembly and activity of oxidative phosphorylation (OXPHOS) complexes I/III, leading to defects in electron transport, reduced ATP production, and impaired energy supply to the heart. Simultaneously, abnormal complex formation affects the stability of the supercomplex, thereby disrupting the inner mitochondrial membrane structure and cristae. Inefficient electron transport causes electron leakage and excessive reactive oxygen species (ROS) generation, triggering oxidative stress and damaging mtDNA. Persistent respiratory chain dysfunction, decreased electron transport efficiency, and ROS accumulation alter mitochondrial membrane permeability, allowing damaged mitochondria to release mtDNA fragments into the cytoplasm. Cytoplasmic mitochondrial DNA functions as a danger-associated molecular pattern (DAMP), activating the cGAS–STING signaling pathway and downstream type I interferons and inflammatory responses. The combined effects of energy deficiency, oxidative stress, and innate immune activation promote cardiomyocyte apoptosis, adverse cardiac remodeling, and persistent systolic dysfunction, ultimately leading to the occurrence and development of heart failure. This comprehensive framework considers mitochondrial DNA mutations as initiating factors, which, through some mechanism, link the loss of mitochondrial bioenergy with inflammatory signaling and heart disease.

## 1 Introduction

Heart failure (HF) is a common end-stage outcome of a variety of cardiovascular diseases. With its high incidence and poor prognosis, it is a major public health challenge facing the world (1). The pathogenesis of HF involves multiple interrelated processes, including cardiac energy metabolism disorders, myocardial damage and chronic inflammation (2–4). Although clinical intervention can improve the prognosis of some patients, there are still many challenges for individuals with hereditary or premature cardiomyopathy.

The heart is a highly energy-dependent organ, and mitochondria play a central role in maintaining the structural integrity of the heart muscle and normal heart function. A large amount of evidence shows that mitochondrial dysfunction is a common feature of myocardial tissue in HF, which is closely related to impaired energy metabolism, enhanced oxidative stress and myocardial cell damage (3, 5), especially in hereditary or idiotic HF. It is worth noting that there are significant individual differences in these changes.

And its upstream regulation mechanism is still not clear. Mitochondrial DNA is a key genetic component of mitochondria and an important subunit encoding the oxidative phosphorylation system. mtDNA mutations have been widely reported in a variety of mitochondrial diseases and cardiomyopathy (3, 6, 7). However, most studies mainly explain the pathogenic effects of mtDNA mutation from the perspective of energy metabolism. This metabolic-centered perspective is not enough to explain the significant phenotypic heterogeneity observed in clinical practice. Individuals with the same or similar mtDNA mutations often have significant differences in disease onset, severity and clinical manifestations.

With the progress of this field, it is increasingly recognized that mtDNA mutation load and heterogeneity levels play a crucial role in the degree of mitochondrial dysfunction and the diversity of disease manifestations (8, 9). However, in most studies and reviews related to HF, mtDNA heterogeneity is still mainly regarded as background information, rather than being systematically included in the core variables to explain pheterogeneity. With the deepening of our understanding of mtDNA biology, more and more evidence shows that mtDNA released by damaged mitochondria can act as a damage-related molecular pattern (DAMP) to activate the inherent immune signaling pathway of cGAS-STING, thus causing chronic inflammation and Promote myocardial structural remodeling (10–13). These findings suggest that the pathological effects of mtDNA mutations are not limited to energy metabolism defects, and the interaction between metabolic imbalance and immune activation may further amplify its effects during the progression of HF.

Based on this, this review focuses on mtDNA mutation and heterogeneity in myocardial cells in HF, and systematically explains its pathological role in the occurrence and development of diseases, including mitochondrial dysfunction, oxidative stress and immune activation. By integrating the intrinsic link between metabolic imbalance and inflammatory immune response, we aim to provide a mechanism framework to explain the significant molecular and clinical heterogeneity of HF, and explore potential diagnosis and treatment strategies for the functional state of mtDNA.

## 2 Clinical manifestations and diagnosis of cardiomyopathy

### 2.1 Clinical manifestations of cardiomyopathy

Mitochondrial diseases are rare genetic disorders arising from mutations in nuclear DNA or mitochondrial DNA (mtDNA), resulting in mitochondrial dysfunction. Approximately one-third of patients with mitochondrial diseases progress to some form of cardiomyopathy, exhibiting marked clinical heterogeneity and a broad spectrum of severity ranging from asymptomatic to life-threatening arrhythmias and HF, frequently accompanied by multisystem involvement (7). HF is the main feature of mitochondrial cardiomyopathy. In cardiomyopathy associated with mtDNA m.3243A>G mutation, the incidence of congestive HF (CHF) is about 35%, and the average age of onset is about 51.3 years old (14). The most common symptom of HF is dyspnea, which is also the primary reason for medical consultation. One reported case involved a 51-year-old patient presenting with dyspnea; echocardiography revealed a markedly reduced left ventricular ejection fraction of 26% (normal range: 55%–70%), leading to a diagnosis of HF with reduced ejection fraction (15).

In addition to HF, patients with mtDNA mutations display a constellation of multisystem impairments that form distinctive clinical syndromes. Diabetes mellitus and sensorineural hearing loss represent the two most prevalent associated features, affecting approximately 49% and 63% of patients, respectively (14). This association has been confirmed in clinical case studies. The study found that all patients with m.3243A>G mutation in cardiomyopathy showed myocardial hypertrophy, juvenile-pathogenic diabetes and hearing loss (16). These observations show that mtDNA mutations give priority to high-energy-dependent tissues, including myocardial cells, pancreatic islet beta cells and cochlear hair cells. In addition to the above manifestations, some patients may also have exocrine dysfunction of the liver, kidney and pancreas, which are particularly pronounced in Pearson's syndrome (17).

### 2.2 Imaging and histological features

Echocardiography is a non-invasive technology that can effectively assess the structural and functional abnormalities of the heart. In patients with mitochondrial cardiomyopathy caused by m.3243A>G mutation, echocardiography results often show cardiac hypertrophy, which usually reflects the change from hypertrophic cardiomyopathy to dilated cardiomyopathy (16, 18). Doppler echocardiography can further evaluate diastolic function and contractile function. Given that mtDNA-associated cardiomyopathy can develop dysfunction in the early stage, echocardiography evaluation provides valuable information for the early detection and progression evaluation of the disease.

Electron microscope can directly observe the mitochondrial morphology in myocardial cells (19). Mitochondrial crest abnormalities, including the formation of onion-like lesions, are associated with impaired heart function, and the severity of mitochondrial structural changes is positively related to the degree of cardiac dysfunction (16). Importantly, changes in the ultrastructure of mitochondria usually preced the appearance of clinical symptoms, which highlights the value of electron microscopy as an early diagnostic tool (20).

Histological examination also serves as an early detection method for mtDNA-related cardiomyopathy. Masson's trichrome staining can reveal disorganized myocardial fiber arrangement and interstitial fibrosis (21), while combined succinate dehydrogenase (SDH) and cytochrome c oxidase (COX) double-labeling histochemistry specifically identifies regions of mitochondrial respiratory dysfunction (22). In mtDNA deletion syndromes, affected skeletal muscle typically shows a mosaic or patchy distribution of COX-negative/SDH-positive fibers, reflecting intercellular differences in mtDNA mutation load. When mutation load exceeds the functional threshold, cytochrome c oxidase activity is impaired and mitochondrial proliferation occurs as compensation, resulting in enhanced SDH staining.

It should be noted that although histopathological examination typically requires invasive procurement of muscle or myocardial biopsy specimens, it retains substantial clinical value in the diagnosis of mtDNA-related diseases. First, histopathology allows direct assessment of the spatial distribution of mitochondrial dysfunction at the tissue level—for example, the mosaic pattern of COX-negative/SDH-positive fibers—which is regarded as a key phenotypic manifestation of mtDNA heteroplasmy in tissue. Second, compared with molecular testing of peripheral blood, tissue samples more accurately reflect mutation load and functional impairment in the target organ, thereby enhancing diagnostic specificity. Furthermore, histopathological analysis can be combined with electron microscopy to evaluate mitochondrial ultrastructure and supplemented with immunohistochemical and enzyme histochemical techniques to provide a comprehensive evaluation of disease severity and progression.

### 2.3 Advances in molecular genetic diagnostic technologies

High-throughput sequencing can simultaneously detect large numbers of gene sequences and identify low-frequency mutations. Researchers have used this technology to discover mutations in the mitochondrial gene *MT-RNR2* in patients with hypertrophic cardiomyopathy and validated through cellular models that the stability of the mitochondrial inner membrane GTPase OPA1 contributes to cardiac hypertrophy (23, 24). Gene chip technology, due to its high-throughput and high-precision features, is widely used for large-scale screening of mtDNA mutations. In a study of patients with coronary artery disease (CAD), researchers identified 25 mtDNA nucleotide variations using gene sequencing, including 10 missense mutations, 9 synonymous polymorphisms, and 6 tRNA gene variations, and reported for the first time that the m.8231C>A mutation is associated with CAD (25). Single-cell sequencing technology further enhances detection sensitivity, revealing the heteroplasmy of mtDNA mutations in tissues, such as identifying the mt-tRNA^Ser^(AGY) 12265A>G mutation and mt-tRNA^Cys^ 5821G>A (26).

In summary, the clinical manifestations of mtDNA mutation-associated cardiomyopathy are extremely complex: patients harboring the same mutation may remain in a long-term subclinical state or progress to severe HF. This phenotypic variability is largely attributable to differences in mtDNA heteroplasmy levels and threshold effects. Therefore, the following sections will focus on the characteristics of mtDNA heteroplasmy and its phenotypic consequences in HF.

## 3 mtDNA mutations and heart failure

### 3.1 mtDNA point mutations

mtDNA point mutations are closely linked to the development of HF. The m.3243A>G mutation is the most common pathogenic mutation, located in the *MT-TL1* gene, primarily affecting the stability and functional integrity of tRNA-Leu (UUR). This mutation will reduce the translation efficiency of mitochondrial proteins, which will eventually affect the energy metabolism of myocardial cells (27). In clinical studies, patients with m.3243A>G mutations usually show three main symptoms: cardiac hypertrophy, diabetes and hearing loss. Under electron microscopy, cardiomyocyte mitochondria in these patients display disordered cristae structures, onion-like lesions, etc., with the degree of mitochondrial lesions positively correlating with cardiac dysfunction (16). Studies have found that the m.8231C>A mutation significantly increases the risk of CAD, potentially involving increased oxygen species ROS production and oxidative phosphorylation dysfunction (25).

The impact of mtDNA point mutations on cardiac function can be summarized in three principal aspects. First, destabilization of tRNA-Leu (UUR) impairs synthesis of key respiratory chain complex I, III, and IV subunits (28). Second, the mutation induces mitochondrial morphological changes—including swelling and cristae disruption—while weakening mitochondria-associated endoplasmic reticulum membrane (MAM) interactions, thereby disrupting calcium homeostasis and directly promoting cardiomyocyte hypertrophy (27). Third, progressive mutation accumulation overwhelms mitochondrial quality control systems, reducing clearance of damaged organelles and establishing a vicious cycle of worsening dysfunction. In a mouse model harboring the *ND6* G13997A mutation, defective reverse electron transport (RET)-associated reactive ROS production leads to diastolic dysfunction and impaired exercise tolerance (29).

The m.3243A>G mutation is often closely associated with dilated cardiomyopathy and hypertrophic cardiomyopathy. Japanese researchers studied three related cases and found that mutation load positively correlates with the degree of cardiac involvement: a 43-year-old male patient with a shorter disease course showed no obvious cardiomyocyte hypertrophy, while patients with a disease course exceeding 20 years exhibited significant cardiac hypertrophy and other severe cardiac lesions (16).

### 3.2 mtDNA heteroplasmy

mtDNA heteroplasmy refers to the coexistence of wild-type and mutant mtDNA molecules within a single cell, with the proportion of mutant mtDNA being a key factor determining the clinical manifestations of mitochondrial diseases (30). The presence of mtDNA heterogeneity contributes to considerable variability in the clinical manifestations of HF patients. Diseases associated with the m.3243A>G mtDNA mutation exhibit a broad spectrum, ranging from mitochondrial encephalopathy, lactic acidosis, and stroke-like episodes (MELAS) to diabetes and HF, with varying degrees of severity (14). Even in patients with the same pathogenic mtDNA mutation, there are significant differences in cardiac involvement, age of onset and incidence of HF, which reflects significant clinical heterogeneity, mainly due to changes in the level of mtDNA heterogeneity in different tissues (30).

**Figure 1:**
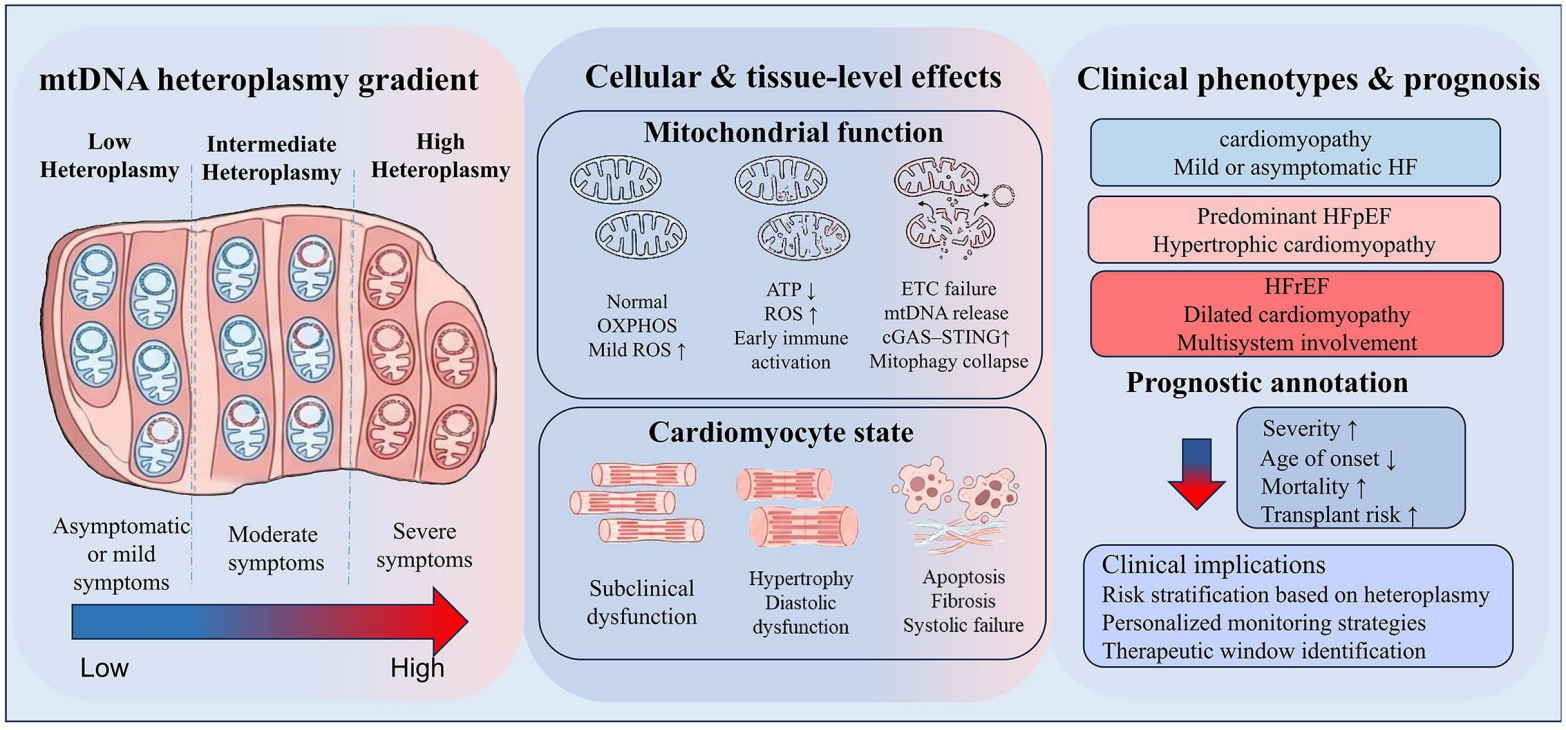
Association between mtDNA heteroplasmy and mitochondrial dysfunction, myocardial injury, and clinical phenotypes. This figure illustrates the progressive impact of increasing mtDNA heterogeneity on cardiomyocyte dysfunction, myocardial remodeling, and clinical phenotype. At low levels of heteroplasmy, OXPHOS is largely preserved, resulting in only mild or subclinical functional impairment. Moderate heterogeneity is characterized by reduced ATP production, increased ROS and early immune activation, which promotes myocardial cell hypertrophy and diastolic dysfunction. When heterogeneity is high, ETC dysfunction, mtDNA release and abnormal activation of cGAS-STING lead to mitochondrial autophagy dystosis, myocardial cell apoptosis, fibrosis and contractile dysfunction. These cell changes correspond to different clinical phenotypes: low heterogeneity is usually asymptomatic or mild symptoms; moderate heterogeneity is associated with hypertrophic cardiomyopathy and ejection fraction retention heart failure (HFpEF); high heterogeneity is associated with dilated cardiomyopathy, ejection fraction reduction heart failure (HFrEF) and multi-system Related to suffering. In short, the model emphasizes that mtDNA heterogeneity is a potential determinant of the severity of the disease, and it is also a tool for risk stratification and individualized management of cardiomyopathy.

Studies have demonstrated a close association between mtDNA heteroplasmy and disease severity, age of onset, and prognosis. The mtDNA heteroplasmy level in specific organs, particularly the liver, shows a significant negative correlation with the patient's age at death; higher mutation proportions correspond to earlier ages at death (14). For the heart, mtDNA heteroplasmy level is central to determining disease penetrance and the intensity of clinical manifestations. A large-scale whole-genome sequencing study in a clinically unselected population confirmed that pathogenic mtDNA variants have low penetrance in such populations, but m.3243A>G is an exception when heteroplasmy is high (31). When heteroplasmy of m.3243A>G reaches or exceeds 10% (defined as the percentage of mutant mtDNA relative to total mtDNA copy number in the sample), the risk of multisystem disease—including diabetes mellitus, deafness, and HF—increases markedly. mtDNA mutations also display pronounced inter-individual variability: when mutation load surpasses the bioenergetic threshold, cardiomyocytes exhibit significantly reduced ATP production and aberrant calcium signaling, leading to impaired cardiac function. Nevertheless, due to individual differences, some patients with high-level mtDNA mutations remain free of overt cardiac dysfunction (32) (Figure 1).

In summary, elevated mtDNA heteroplasmy levels indicate increased disease risk, yet their influence on cellular or tissue phenotypes is not deterministic. Consequently, heteroplasmy level serves as a key parameter for assessing disease risk and progression trajectory, rather than a definitive predictor of clinical outcome. Quantitative assessment of mtDNA heteroplasmy in myocardial or other tissues helps explain the marked phenotypic heterogeneity observed in HF and provides substantial clinical value for evaluating cardiomyopathy severity and prognosis.

## 4 mtDNA mutations and mitochondrial function

### 4.1 Respiratory chain complex abnormalities and energy metabolism disorders

mtDNA point mutations can lead to respiratory chain dysfunction. These mutations directly affect the encoding of key subunits in the electron transport chain (ETC), preventing normal complex assembly, reducing oxidative phosphorylation (OXPHOS) efficiency, and leading to decreased or lost ATP synthesis capacity (33). The m.3243A>G mutation specifically disrupts normal respiratory chain activity (34). The heart is a high-energy-consuming organ, and respiratory chain dysfunction has a more pronounced impact on it.

In addition to point mutations, large-scale mtDNA deletions also affect respiratory chain function. In patients with progressive external ophthalmoplegia (PEO) and Kearns-Sayre syndrome (KSS), mtDNA deletions involving multiple respiratory chain complex subunits and tRNA genes can be detected, and the deletion length is closely related to the severity of clinical phenotypes (35). Similarly, the POLG gene mutation encoding mtDNA polymerase *γ* will lead to the deletion of the mitochondrial respiratory chain complex I and disturb the NAD+/NADH ratio, thus exacerbating the overall dysfunction of the mitochondria (36). These genetic defects can impair the function of the electron transfer chain (ETC) and significantly reduce the efficiency of ATP synthesis.

mtDNA mutations that impair the activity of respiratory chain complexes will not only destroy the ETC function, but also change the energy metabolism of cells (37). Myocardial cells need a large amount of ATP to maintain normal function. When the ETC complex I or III is damaged, the energy in the heart will be exhausted in a few seconds, thus triggering the transformation of metabolism from oxidative phosphorylation to glycolysis. This metabolic transformation will significantly reduce the production of ATP, fail to meet the high energy needs of the heart muscle, and lead to cell damage (38). Consequently, respiratory chain dysfunction driven by mtDNA mutations compromises cardiomyocyte energy supply, leading to cardiac functional impairment and promoting HF progression.

### 4.2 Impact of mitochondrial structural abnormalities on cardiomyocytes

In patients with mtDNA mutation-associated cardiomyopathy, abnormal mitochondrial cristae and onion-like or disorganized inner membrane structures are commonly observed (16). These structural alterations are not merely incidental morphological changes but are intimately linked to mtDNA integrity and respiratory chain assembly. Mutation-induced mitochondrial structural abnormalities arise through two main mechanisms. First, pathogenic mtDNA variants (e.g., m.3243A>G in *MT-TL1* and m.3460G>A in *MT-ND1*) impair mitochondrial translation and disrupt OXPHOS complex assembly (39, 40). Defective complex formation destabilizes respiratory chain supercomplexes embedded in the inner mitochondrial membrane (IMM), whose architecture is tightly coupled to cristae organization. Loss of supercomplex stability alters membrane curvature and cristae junction integrity, ultimately resulting in cristae rarefaction and structural remodeling (41). Second, large-scale mtDNA deletions have been shown to cause mitochondrial structural disorganization and altered intra-mitochondrial mtDNA distribution. In Pearson syndrome, caused by a single large mtDNA deletion, cell models consistently exhibit rounder, swollen mitochondria with reduced and damaged cristae and electron-lucent matrix (42).

Mitochondrial structural alterations are closely linked to cardiomyocyte hypertrophy and apoptosis. Mutations in the TAZ gene cause cardiolipin remodeling defects that impair mitochondrial function and precipitate cardiomyopathy (43). Extensive evidence shows that mitochondrial internal architecture undergoes marked remodeling during HF, including reduced cristae stacking, which lowers ATP synthesis efficiency and elevates ROS production (44). These abnormalities influence cardiomyocyte function through multiple molecular pathways and exhibit a clear correlation with cardiac performance. Studies have demonstrated that mitochondrial dysfunction in cardiomyocytes occurs almost concomitantly with ventricular repolarization abnormalities; cardiac-specific knockout of the mitochondrial calcium uniporter ameliorates both defects (45). In human induced pluripotent stem cell-derived cardiomyocytes (hiPSC-CMs), DNM1L mutations (encoding dynamin-related protein 1) cause abnormally elongated mitochondria, reduced membrane potential, and sharply decreased ATP output (46). Moreover, structural defects indirectly impair cardiac function by disrupting mitochondria–endoplasmic reticulum coupling (MAMs). Loss of FUNDC1 destabilizes MAM architecture, inhibiting IP3R2-Grp75-VDAC1 complex formation and thereby perturbing calcium signaling and energy metabolism (47). Collectively, these findings indicate that mitochondrial structural abnormalities are not only pivotal drivers of cardiomyocyte dysfunction and death but also key contributors to the variable severity of HF.

In summary, these studies show that mitochondrial structural abnormalities are a direct factor in myocardial cell dysfunction, and the recovery of mitochondrial structure is accompanied by the recovery of function. Therefore, targeted mitochondrial structural remodeling represents a promising strategy for the treatment of HF.

### 4.3 Abnormal ROS production and oxidative stress

mtDNA mutations constitute a critical driver of mitochondrial dysfunction and exaggerated oxidative stress in failing myocardium. These mutations not only promote reactive ROS generation but also allow ROS to inflict further damage on mtDNA, establishing a vicious cycle (48, 49). In cell lines harboring mtDNA mutation, mitochondrial ROS levels are significantly elevated compared with wild-type controls (50). This finding was corroborated by MitoSOX assays in patient-derived iPSC endothelial cell models (51). Similarly, markedly increased ROS production has been documented in m.3460G>A LHON cybrid models and m.11778G>A models (52, 53).

These mutations primarily compromise complex I assembly and electron transfer efficiency, increasing the probability of electron leakage at the FMN site of complex I and the Qo site of complex III. Consequently, electrons prematurely react with molecular oxygen, generating excessive superoxide and other ROS (54). The *ND5* gene is a mutational hotspot in mtDNA; experimental models demonstrate elevated mitochondrial ROS levels (55). *MT-ND5* mutations are also strongly associated with infantile-onset hypertrophic cardiomyopathy (56). The POLG mutator mouse model, driven by accumulated mtDNA mutations, provides compelling mechanistic evidence. Defective proofreading activity of mitochondrial DNA polymerase *γ* leads to progressive accumulation of mtDNA point mutations. These animals develop progressive respiratory chain dysfunction and increased mitochondrial ROS production (57). Affected mice exhibit marked cardiac hypertrophy, ventricular dilation, and systolic/diastolic dysfunction. Crossing with mice overexpressing mitochondria-targeted catalase (mCAT) partially rescues the cardiac phenotype, demonstrating a direct causal role of ROS in mutation-driven cardiomyopathy (58).

When mutation load accumulates beyond the bioenergetic threshold required for respiratory chain assembly, electron leakage and ROS production increase in a nonlinear fashion (59). Lacking histone protection and located in close proximity to the inner membrane, mtDNA is highly susceptible to ROS attack, resulting in 8-oxoguanine oxidative base lesions that reduce replication and transcription fidelity and further elevate mutation load and heteroplasmy (60). Similarly, ROS act on the cardiolipin-rich inner membrane microenvironment; cardiolipin peroxidation directly impairs complex III activity and destabilizes the respiratory chain (61).

Thus, in mtDNA-related HF, oxidative stress is not merely a secondary epiphenomenon but a mutation-driven mechanistic amplifier that establishes the pathological cycle “mtDNA mutation → respiratory chain defect → ROS accumulation → increased mutation load.” This positive-feedback loop converts primary genetic defects into a state of sustained oxidative stress, driving myocardial energy failure and progressive decompensation.

### 4.4 Mitochondral DNA and immune activation

Emerging studies demonstrate significantly elevated circulating mtDNA levels in patients with mitochondrial diseases, accompanied by innate immune activation (62). mtDNA release primarily depends on mitochondrial membrane permeability and occurs via several mechanisms: mtDNA instability (63), excessive ROS production (64), mitochondrial membrane depolarization, and opening of the mitochondrial permeability transition pore (mPTP) (65). Although mtDNA mutations do not directly disrupt membrane integrity, multiple pathogenic variants have been shown to induce respiratory chain dysfunction, reduced electron transfer efficiency, ROS accumulation, and oxidative damage, thereby priming mitochondria for permeability transition. In LHON cybrid models carrying m.11778G>A, increased ROS and mitochondrial depolarization have been documented, indicating a functional imbalance permissive for membrane permeabilization (53). In PolG mutator mice lacking 3′–5′ exonuclease proofreading activity—characterized by cardiomyopathy and hearing loss—cytosolic mtDNA is detectable in MEF cells and activates cGAS/STING-dependent IFN-β expression (66). Direct mutation-specific evidence remains limited; current data suggest that mtDNA release in mutation carriers is predominantly mediated by secondary bioenergetic collapse and membrane permeability transition rather than direct structural consequences of the mutation itself.

Originating from bacteria, mtDNA shares structural features with microbial DNA and, upon cytosolic release, functions as a damage-associated molecular pattern (DAMP) (67). It is recognized by cGAS, which catalyzes cGAMP synthesis and activates the STING pathway to trigger innate immune responses (11). STING subsequently recruits TBK1, activating NF-κB and inducing expression of pro-inflammatory cytokines such as TNF and IL-1β (68).

Although the molecular mechanisms of cytosolic mtDNA activating the cGAS-STING pathway have been extensively characterized in immune cells, infection models, and multi-tissue inflammation systems, cardiovascular-specific validation has only recently begun using cardiac models. For instance, MFN2 downregulation in ischemia–reperfusion injury leads to cytosolic mtDNA accumulation and STING activation, indicating functional relevance in the cardiac microenvironment. In diabetic myocardial ischemia–reperfusion injury, reduced mitochondrial fusion leads to cytosolic mtDNA accumulation and cGAS-STING activation (69). However, direct cardiomyocyte-specific models based on pathogenic mtDNA mutations remain scarce. Evidence from systemic STING-knockout models and immune-cell-mediated inflammation suggests that the mtDNA–cGAS–STING axis in HF involves both cardiomyocyte-autonomous responses and secondary amplification by immune cells (70).

Myocardial inflammation exerts well-documented amplifying effects during HF progression. Activation of the cGAS-STING pathway promotes NLRP3 inflammasome assembly and IL-1β release, further intensifying the inflammatory response (71). Although NLRP3 inflammasome activation has been shown to participate in myocardial remodeling in pressure-overload and injury models, evidence that mtDNA directly triggers this process remains largely indirect, necessitating further elucidation of its cardiac regulatory mode. Moreover, released mtDNA can directly engage and activate the NLRP3 inflammasome (NOD-, LRR-, and pyrin domain-containing protein 3), eliciting inflammatory responses (72). These innate immune processes are particularly critical in HF: mtDNA release from cardiomyocytes activates inflammatory signaling, establishing a low-grade yet persistent inflammatory state that provides sustained stimuli for myocardial fibrosis and ventricular remodeling (73). However, the temporal dynamics of mtDNA release and its causal relationship with cardiomyocyte dysfunction require further clarification. While cytosolic mtDNA activates intracellular DNA-sensing pathways, extracellular mtDNA engages Toll-like receptor 9 (TLR9) on innate immune cells (13).

Abnormal mtDNA release converts mitochondrial damage into persistent inflammatory signaling via innate immune activation, thereby accelerating HF progression. These insights provide a theoretical foundation for developing novel immunometabolic therapies for HF. Current evidence indicates that the process encompasses both cardiomyocyte-autonomous inflammatory responses and systemic amplification mediated by immune cells. Future establishment of cardiomyocyte-specific models will enable precise delineation of the cellular sources and signaling hierarchy of mtDNA-driven immune activation, ultimately advancing precision therapeutic strategies for HF based on immunometabolic regulation.

### 4.5 Mitochondrial dynamics imbalance and myocardial pathological changes

Pathological Cardiac Remodeling Mitochondrial fusion and fission are essential for maintaining quality control and energy homeostasis. Recent studies indicate that respiratory chain dysfunction and excessive ROS production secondary to mtDNA mutations indirectly disrupt the fusion–fission balance (74, 75). In cellular models carrying complex I mutations (e.g., LHON m.11778G>A) or *ND* gene defects, impaired electron transfer leads to sustained ROS elevation (52, 53). The primary regulator of mitochondrial fission is Drp1; elevated ROS upregulates Drp1 expression while suppressing fusion proteins (MFN1/2 and OPA1), resulting in excessive fission and mitochondrial fragmentation (76). Moreover, ROS influences Drp1 at both transcriptional and post-translational levels, promoting Ser616 phosphorylation and outer-membrane translocation, thereby driving fission (77). Although direct evidence that specific mtDNA point mutations independently drive dynamic remodeling is currently lacking, ROS-dependent Drp1 activation has been consistently observed across multiple complex I-deficient models. It is therefore reasonable to propose that, in cardiomyocytes, when mutation load exceeds the energy homeostasis threshold, oxidative stress serves as the critical mediator linking genetic defects to dynamic imbalance.

**Figure 2:**
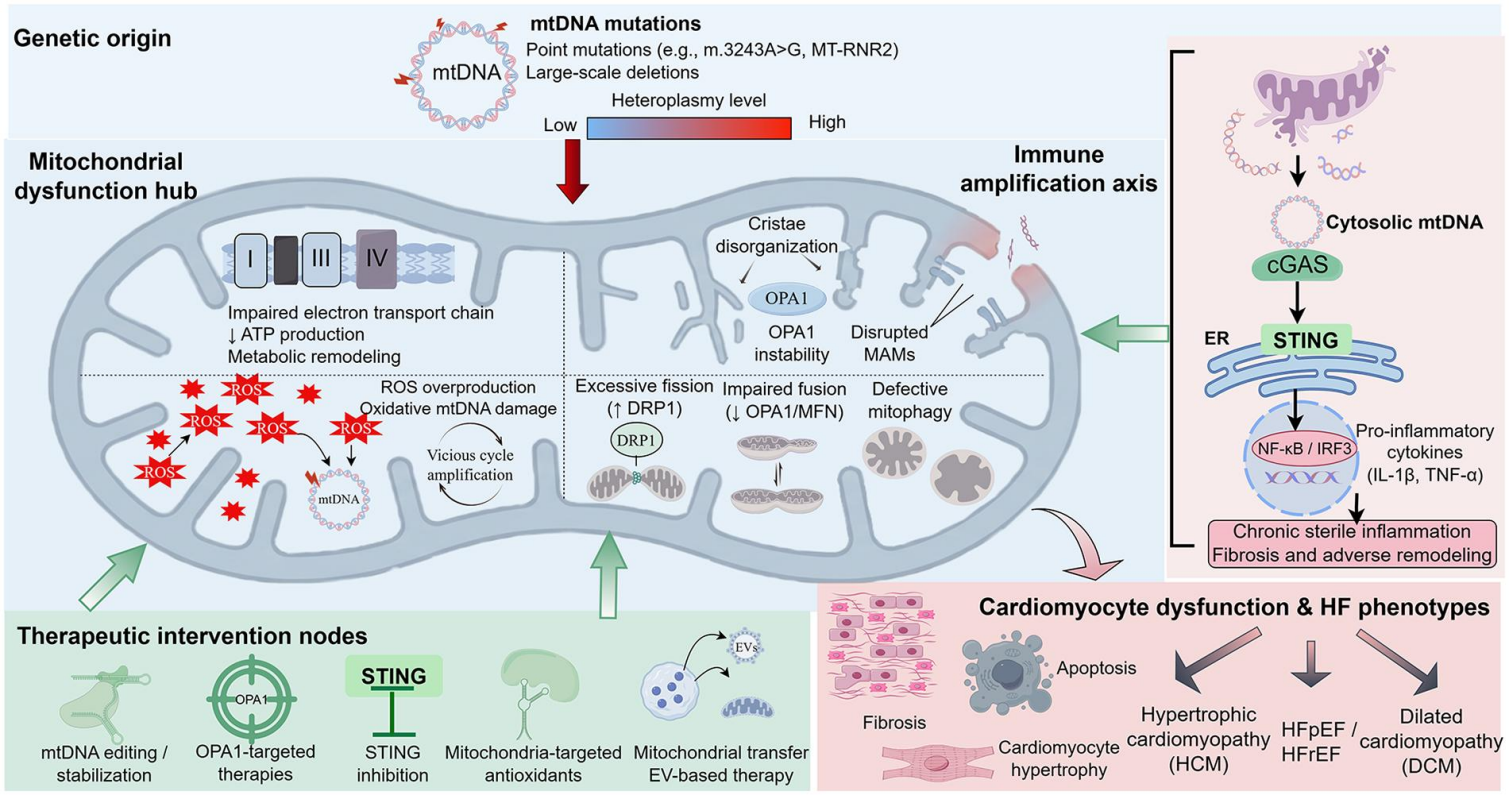
The molecular mechanism and treatment strategy of heart failure caused by mitochondrial DNA mutation. mtDNA mutations mainly include point mutations and large fragment deletions, which are characterized by different levels of heterogeneity. Once the heterogeneity level exceeds the critical threshold, these mutations will destroy the mitochondrial homeostasis, eventually leading to myocardial cell dysfunction. In terms of mechanism, impaired ETC activity will lead to a decrease in ATP generation, accompanied by excessive production of ROS and oxidative damage of mtDNA, thus forming a vicious circle. Concurrently, mitochondrial structural and dynamic abnormalities—including cristae disorganization, OPA1 instability, excessive DRP1-mediated fission, impaired fusion, disrupted mitochondria–associated membranes (MAMs), and defective mitophagy—further exacerbate mitochondrial dysfunction. Mitochondrial membrane damage will promote the release of mitochondrial DNA into cytoplasmic sols, thus activating the cGAS-STING signaling pathway and the production of downstream NF-κB/IRF3-mediated pro-inflammatory cytokines (including IL-1β and TNF-α). This chronic aseptic inflammatory reaction will exacerbate heart remodeling, leading to myocardial cell hypertrophy and fibrosis, and in the late stage, resulting in apoptosis, eventually producing a variety of heart failure phenotypes, including hypertrophic cardiomyopathy (HCM), dilated cardiomyopathy (DCM) and ejection fraction retention heart failure (HFpEF) and reduced ejection fraction heart failure (HFrEF). It also focuses on the potential treatment intervention points for mitochondrial DNA stability, mitochondrial dynamics, oxidative stress, immune signal transduction and mitochondrial substitution strategies.

Mitochondrial dynamic imbalance is tightly coupled to cardiomyocyte calcium homeostasis disruption. Excessive Drp1-mediated fission induces calcium overload, activating the calcium-dependent protease calpain and exacerbating cardiomyocyte injury (78). Loss of the fusion protein Mfn2 destabilizes mitochondria–endoplasmic reticulum contact sites (MAMs), impairing calcium signaling and ultimately causing contractile dysfunction (79). Dynamic dysregulation is also implicated in myocardial fibrosis and pathological hypertrophy, further aggravating HF progression (80) (Figure 2).

This chapter has systematically reviewed the role of mtDNA mutations in HF pathogenesis across five key aspects of mitochondrial biology: energy metabolism, structure, oxidative stress, immune activation, and dynamics. mtDNA point mutations and large deletions directly compromise respiratory chain complex architecture and function, reducing OXPHOS efficiency and placing cardiomyocytes in a state of chronic energy deficit. Cristae disorganization and calcium signaling defects further impair contractile function and accelerate cardiac decompensation. Respiratory chain impairment also drives aberrant ROS generation, causing sustained oxidative damage to lipids, proteins, and mtDNA itself and establishing a vicious cycle. Cytosolic mtDNA acts as a DAMP, activating cGAS–STING and other innate immune pathways to induce chronic inflammation that promotes myocardial fibrosis and ventricular remodeling. At the level of mitochondrial dynamics, imbalanced fusion and fission exacerbate energy metabolism deficits and calcium dysregulation, driving pathological cardiac remodeling.

## 5 Therapeutic strategies for HF related to MtDNA mutations

### 5.1 Traditional drug therapy and supportive therapy

Traditional drugs have not been very effective in treating mtDNA mutation-related HF. Traditional drugs such as diuretics, β-blockers, angiotensin-converting enzyme inhibitors (ACEIs), angiotensin receptor-neprilysin inhibitors (ARNIs), and aldosterone receptor antagonists are widely used in clinical treatment and can improve the prognosis of conventional HF patients, but their efficacy is very limited in patients with mtDNA mutation-related HF. Studies have confirmed that among 14 patients with the *MT-TL1*m.3243A>G mutation, even after receiving standard anti-HF treatment, 10 died from HF combined with multi-organ failure within 5 years of diagnosis (18). Although traditional drugs can improve hemodynamics, they cannot ameliorate defects in the mitochondrial electron transport chain. Genetic analysis in end-stage cardiomyopathy patients revealed that 44.3% may have pathogenic cardiomyopathy gene variants, implying that traditional treatments may have poorer efficacy (81). This situation compels us to find new therapeutic strategies to improve clinical symptoms in HF patients with mtDNA mutations.

Metabolic regulators may be more effective in improving symptoms in patients with mtDNA mutations and can serve as a therapeutic strategy. Studies have found that malonate has a certain protective effect on the heart, inhibiting succinate dehydrogenase (SDH) activity to extend the window for proliferation and regeneration of neonatal mouse cardiomyocytes. After myocardial infarction, it can still promote cardiomyocyte proliferation and vascular regeneration in adult mice (82). The mechanism of malonate may involve metabolic reprogramming and mitochondrial function improvement. The Glu504Lys polymorphism in the aldehyde dehydrogenase 2 (ALDH2) gene is closely related to myocardial ischemia-reperfusion injury, and leukotriene C4 (LTC4) receptor antagonists can alleviate this symptom and improve normal cardiac function (83). Collectively, these studies demonstrate that metabolic modulators can effectively alleviate clinical symptoms in mtDNA mutation-associated HF and warrant consideration as a clinical therapeutic option.

For therapeutic strategies addressing oxidative damage to the heart caused by excessive ROS production, antioxidants such as coenzyme Q10 are commonly used clinically. Although widely used, they lack specificity for mtDNA mutations. Mitochondria-targeted antioxidants, such as cell-penetrating peptides (CPPs), can correct abnormal mitochondrial protein expression and restore mitochondrial function (84). 1-Deoxynojirimycin (DNJ) can repair mitochondrial cristae structural abnormalities caused by *MT-RNR2* mutations by stabilizing the mitochondrial inner membrane GTPase OPA1, providing a new direction for cardiomyopathy treatment (24). In summary, conventional antioxidants show limited efficacy in clinical practice, underscoring the urgent need for novel mitochondria-targeted antioxidants to improve clinical outcomes in HF.

### 5.2 Innovative therapies targeting mitochondrial function

Mitochondrial Function Innovative therapies targeting mitochondrial function are considered highly promising strategies in the HF treatment landscape, as they directly address cardiomyocyte energy metabolism defects. Based on current research progress and clinical translational readiness, these approaches can be broadly categorized into mitochondria-targeted delivery technologies, extracellular vesicle-based therapies, and mitochondrial transplantation at varying stages of development.

Mitochondria-targeted delivery technologies are currently regarded as among the approaches closest to clinical application. For example, mitochondria-penetrating peptides efficiently deliver therapeutic molecules into the mitochondrial matrix, enabling precise intervention in mitochondrial dysfunction (85). The MITO-Porter system is a novel drug delivery platform capable of targeted mitochondrial delivery of therapeutic agents (86). In the context of HF, such targeted delivery strategies can be used to enhance mitochondrial antioxidant capacity (87). Nevertheless, their long-term safety and dose optimization in patients with mtDNA mutations require systematic clinical validation.

In recent years, extracellular vesicle-mediated mitochondrial regulation strategies have gained increasing attention and are considered to possess moderate translational potential. Studies have shown that mesenchymal stem cell-derived extracellular vesicles promote mitophagy and ameliorate inflammation secondary to mitochondrial dysfunction (88). Mitochondria-rich extracellular vesicles (M-EVs) can transfer functional mitochondria to cardiomyocytes, restoring intracellular ATP production and significantly improving cardiac function after myocardial infarction (89). However, clinical translation still faces challenges including standardized preparation, dose consistency, and long-term safety assessment.

By contrast, mitochondrial transplantation represents a highly innovative yet still exploratory therapeutic modality whose principle involves using functional mitochondria to repair dysfunctional ones within cells. Relevant clinical studies have demonstrated that isolation and transplantation of functional mitochondria into cardiomyocytes successfully ameliorates myocardial ischemia-reperfusion injury (90). This approach has also been validated in animal models, where intracoronary delivery of mitochondria to the heart in rabbits confirmed the feasibility of mitochondrial transplantation (91). In cases of mtDNA mutation-induced mitochondrial dysfunction, mitochondrial transplantation can similarly restore function and ATP production (92).

In summary, although preclinical results for these innovative therapies in mtDNA mutation-associated HF are encouraging, significant gaps remain before they can be translated into routine clinical practice. Nevertheless, they offer important insights for the clinical management of mitochondrial dysfunction-related diseases and provide patients with entirely new therapeutic options.

### 5.3 Immune modulation and anti-inflammatory treatment

The development of HF is frequently accompanied by persistent, difficult-to-resolve chronic inflammation (93). Immunometabolic reprogramming serves as the critical nexus linking metabolic dysregulation and inflammatory responses. Ischemic injury and metabolic stress drive systemic inflammatory reactions that promote infiltration of innate and adaptive immune cells into myocardial tissue (4). These immune cells can further undergo metabolic shifts between oxidative phosphorylation and anaerobic glycolysis, leading to the release of either pro-inflammatory or anti-inflammatory mediators (94). It follows that modulation of immune-inflammatory pathways represents a key interventional direction for delaying HF progression. Based on current research advances, immunomodulatory therapeutic strategies also exhibit varying stages of clinical translation.

Targeted therapies against inflammatory cytokines represent one major category. Cytosolic release of mtDNA activates the cGAS-STING signaling pathway, inducing type I interferons and pro-inflammatory cytokine production, which may exacerbate HF symptoms (95). For instance, tumor necrosis factor-α (TNF-α) blockade in chronic HF has been associated with multiple adverse effects (96). Current strategies targeting pro-inflammatory cytokines include interleukin-6 (IL-6) blockade, which has shown therapeutic potential in other inflammatory diseases, although its applicability in cardiovascular disorders requires further clinical investigation (97). Galectin-1 (Gal-1), which regulates T- and B-cell activation, is implicated in HF-related inflammation and represents a potential future target (98). Interleukin-35 (IL-35) has demonstrated cardioprotective effects in various cardiovascular disease models, making IL-35-targeted strategies highly valuable for cardiovascular research (99). Although Gal-1 and IL-35 exhibit cardioprotective actions in experimental models, they remain largely at the preclinical stage. Notably, many current anti-inflammatory strategies derive from systemic inflammation models rather than cardiomyocyte-specific mtDNA mutation models, which may limit mechanistic precision for cardiac-targeted therapies. These studies suggest that future anti-inflammatory approaches should focus on precise inhibition of specific pro-inflammatory cytokine release or blockade of their downstream signaling to mitigate cardiac injury and open new avenues for HF treatment.

Immunomodulators and natural medicines constitute another important category. To alleviate immune responses in HF, the application prospects of immunomodulators are expanding. Certain traditional Chinese medicines have demonstrated unique value in restoring immune balance. For example, Qiliqiangxin (QLQX) capsules were shown in trials for viral dilated cardiomyopathy to downregulate pro-inflammatory cytokines such as IFN-γ, IL-17, and TNF-α while upregulating IL-10, thereby improving cardiac function (100). Matrine also possesses immunomodulatory potential: it inhibits dendritic cell maturation, downregulates the NF-κB pathway, and upregulates ERK1/2 signaling, reducing inflammatory cell infiltration and oxidative damage; moreover, combination with tacrolimus exerts synergistic effects in preventing acute rejection after heart transplantation (101). Alkaloids and polysaccharides from Fuzi (Aconiti Lateralis Radix Praeparata) have likewise been reported to exert anti-inflammatory and immune-regulatory effects (102). Although traditional medicines offer multi-target advantages, their complex mechanisms and limited standardization necessitate further validation through high-quality randomized clinical trials.

Cell-and neuro-immunomodulatory strategies extend beyond the aforementioned approaches. Mesenchymal stem cell-derived extracellular vesicle therapy is safer and more effective, as demonstrated in multiple cardiovascular disease models where it reprograms macrophages toward an anti-inflammatory phenotype and improves symptoms (103). Exosomes from endogenous immune cells and exogenous stem cells can modulate immune responses and promote repair of infarcted cardiomyocytes (104). In addition to cell-based therapies, neural pathways also regulate immunity. The vagus nerve has been shown to exert immunomodulatory effects and may hold therapeutic potential in HF (105). Modulation of autonomic nervous system balance is likewise considered beneficial for alleviating inflammation and improving long-term cardiovascular outcomes, although trial results in HF remain inconsistent and require further investigation (106). Neuro-immunomodulatory strategies are gaining attention; for example, vagus nerve stimulation has demonstrated anti-inflammatory potential, yet its clinical efficacy in HF remains controversial and needs additional validation through clinical studies.

Overall, from a translational medicine perspective, immunomodulatory pharmacotherapy represents the most immediately feasible strategy, followed by mitochondria-targeted antioxidant delivery. In contrast, mitochondrial transplantation and gene editing remain long-term, high-risk approaches that require further mechanistic and safety validation.

## 6 Risk stratification and precision therapy translation strategies for mtDNA

### 6.1 Biomarkers for prognosis and prognostic assessment

Precise diagnosis and risk stratification of HF have long been limited by the lack of biomarkers that combine high sensitivity and specificity. The fundamental reason for this bottleneck is that existing markers predominantly reflect already-established myocardial injury rather than the early molecular drivers of disease onset. Given the close association between mitochondrial dysfunction and HF development, mitochondrial-derived molecular signals hold greater potential for early disease assessment.

mtDNA, as a DAMP, can be released into the bloodstream during subclinical myocardial injury or mitochondrial stress phases, with its level changes not entirely dependent on overt cardiac dysfunction. Therefore, we posit that mtDNA is more suitable as a pre-onset risk prediction signal for HF, rather than merely a passive reflection post-disease occurrence. Accumulating evidence indicates that circulating mtDNA levels reflect early mitochondrial injury, energy metabolic imbalance, and a state of low-grade immune activation, thereby providing a potential means to identify high-risk individuals who have not yet progressed to overt HF (73). Importantly, the clinical value of mtDNA is not limited to changes in its cyclic abundance. Specific pathogenic mtDNA mutations, such as m.3243A>G, constitute the molecular basis of some patients' diseases, and their identification helps to clarify the cause and accelerate the diagnosis process (15). In a word, the quantitative changes and mutation-specific characteristics of mtDNA provide a mechanical connection between molecular damage and clinical phenotypes, so that the management of HF shifts from passive diagnosis to active risk prediction.

However, clinical application of mtDNA still faces critical methodological challenges. First, quantitative detection of mtDNA heteroplasmy is highly dependent on the technical platform. Commonly used methods include high-depth next-generation sequencing, droplet digital PCR (ddPCR), and ultra-sensitive single-molecule sequencing technologies (107, 108). These techniques offer clear advantages in sensitivity and mutation-site resolution, yet inter-platform quantitative consistency remains insufficient. In addition, plasma cell-free mtDNA levels are susceptible to confounding by tissue origin, sample processing methods, and detection time windows, all of which may introduce bias in heteroplasmy assessment (109). Therefore, establishment of unified standards for sample collection, processing, and detection is a key prerequisite for integrating mtDNA heteroplasmy into clinical risk assessment systems. Furthermore, mtDNA heteroplasmy exhibits marked tissue-specific differences. Although clinical testing typically relies on peripheral blood samples, heteroplasmy levels in blood do not fully equate to mutation load in myocardial tissue (110). Future longitudinal cohort studies are therefore needed to clarify the correlation between peripheral circulating mtDNA heteroplasmy and myocardial mutation spectra, thereby enhancing its predictive value in patient stratification.

In addition to mtDNA, proteins and miRNAs closely associated with mitochondrial functional status are increasingly forming more informative biomarker panels. Downstream effector molecules related to impaired mitochondrial OXPHOS, excessive reactive oxygen species generation, and mtDNA mutation accumulation can further enhance prognostic accuracy (80). Specific miRNA combinations have shown promise in disease phenotype stratification and can distinguish HF with preserved vs. reduced ejection fraction (111). These miRNAs frequently participate in mitochondrial quality control and energy metabolism regulation, with expression changes closely reflecting the molecular stage of disease. Combining circulating mtDNA levels with specific miRNA profiles and even mitochondrial function-related protein expression enables construction of multimodal biomarker panels (26).

Integrating circulating mtDNA levels, specific miRNA signatures, and dynamic changes in mitochondrial function-related proteins is expected to construct multimodal biomarker panels, thereby forming a more comprehensive prognostic assessment framework that provides a robust molecular basis for determining disease trajectories, predicting adverse outcomes, and evaluating therapeutic responses.

### 6.2 Clinical translation of innovative therapeutic strategies

mtDNA-based preclinical risk prediction and prognostic stratification provide clear clinical application scenarios for mitochondria-targeted interventions. By identifying the extent of mtDNA damage, mutation load, and associated molecular features, potential beneficiaries can be screened with greater precision, laying a foundation for individualized treatment strategies.

With the advancement of pluripotent stem cell technology, researchers have reprogrammed fibroblasts from patients with mtDNA mutations into induced pluripotent stem cells (iPSCs) and constructed *in vitro* cardiomyocyte models carrying specific mtDNA abnormalities. These models faithfully recapitulate key disease features and enable evaluation of the effects of different interventions on mitochondrial function and myocardial phenotypes (112). Because of mtDNA heteroplasmy, reprogramming can simultaneously generate patient-specific iPSC lines with high or low heteroplasmy levels; only high-heteroplasmy lines exhibit mitochondrial dysfunction and related features (113). Consequently, such models provide a critical experimental platform for predicting treatment responses and screening drugs in high-risk patients.

It is noteworthy that mtDNA heteroplasmy holds potential stratification value in the formulation of precision treatment strategies. Patients with high heteroplasmy are more likely to exhibit respiratory chain dysfunction and may theoretically benefit more from mitochondrial replacement or gene repair approaches, whereas those with low heteroplasmy may respond better to metabolic modulation and anti-inflammatory interventions. However, unified clinical threshold standards are currently lacking, and different mutation sites and tissue types may possess distinct functional damage thresholds—a major factor limiting clinical application.

Such strategies are most likely to first serve specific high-risk subgroups. For gene editing of mtDNA mutations, the novel mitochondria-targeted delivery system RNP-MITO-Porter has achieved precise editing of specific sites in cell models (114), yet it still faces major obstacles including delivery efficiency, off-target risks, and heteroplasmy threshold control. Overall, a significant gap persists between the research enthusiasm for innovative therapies and their real-world translational maturity. Therapeutic strategies targeting mitochondrial metabolism, oxidative stress, and biogenesis, when combined with dynamic monitoring of mtDNA and its downstream molecular markers, hold promise for assessing treatment responses and guiding regimen optimization. Mitochondria-specific antioxidants such as Nrf2 pathway activators can exert cardioprotective effects by alleviating oxidative stress and improving mitochondrial function (115).

Artificial intelligence-assisted multi-omics integration analysis provides new technological support for mtDNA-driven risk assessment and therapeutic decision-making. By integrating quantitative mtDNA data, mutation profiles, transcriptomic, and metabolomic information, more refined risk prediction models can be constructed (116). Furthermore, integration of multi-omics data with heteroplasmy analysis is expected to establish patient stratification algorithms centered on mutation load, thereby guiding prioritization of different therapeutic strategies. For example, patients with high heteroplasmy may be more suitable for mitochondrial replacement or gene repair therapies, while those with low heteroplasmy may benefit more from metabolic modulation or anti-inflammatory interventions. The application of remote monitoring and wearable devices also creates conditions for dynamic evaluation of mitochondria-targeted treatment efficacy. Remote monitoring platforms enable real-time HF surveillance, wearable devices provide continuous heart rate and rhythm monitoring, and implantable devices such as pacemakers allow real-time physiological assessment (117).

Overall, these emerging technologies do not exist in isolation but collectively serve mtDNA-based risk re-stratification and treatment response assessment. By integrating molecular subtyping, risk prediction, and targeted intervention strategies, it is anticipated that mtDNA abnormality-associated heart failure will progressively transition from exploratory research to implementable precision clinical management.

## 7 Conclusion

In summary, mtDNA mutations are not merely a single genetic event in the pathogenesis of heart failure but deeply participate in disease progression from subclinical stages to end-stage heart failure through multilevel, multi-pathway mitochondrial dysfunction. In contrast to the traditional view that attributes myocardial injury solely to energy metabolism disorders, accumulating evidence indicates that mtDNA mutations and their heteroplasmy levels not only determine oxidative phosphorylation efficiency and myocardial energy supply but also drive oxidative stress, disrupt mitochondrial structural homeostasis and dynamic balance, and activate innate immune pathways such as cGAS–STING, thereby forming a continuously amplified pathological loop between metabolic imbalance and chronic inflammation. This mechanistic framework helps explain the widespread clinical phenomenon of phenotypic heterogeneity, whereby patients carrying identical mtDNA mutations exhibit marked differences in age of onset, severity of cardiac involvement, and prognosis.

On this basis, mtDNA heteroplasmy levels and functional status should be regarded as critical bridges connecting molecular injury and clinical phenotypes rather than mere background genetic features. From a diagnostic perspective, integration of quantitative mtDNA changes, mutation spectra, and mitochondrial-related miRNA and protein markers holds promise for early identification of high-risk populations and more refined disease stratification. From a therapeutic perspective, treatment models relying solely on traditional hemodynamic improvement are insufficient to reverse pathological processes driven by mitochondrial dysfunction; interventions targeting mitochondrial metabolism, structural homeostasis, and immune-inflammatory pathways may provide more precise therapeutic avenues for mtDNA mutation-associated heart failure. Future studies urgently require systematic integration of mtDNA heteroplasmy, functional phenotypes, and long-term prognostic data in clinical cohorts to truly translate mitochondrial biology discoveries into precision diagnosis and individualized treatment.
